# Effectiveness of using teachers to screen eyes of school-going children in Satna district of Madhya Pradesh, India

**DOI:** 10.4103/0301-4738.57157

**Published:** 2009

**Authors:** Anand Sudhan, Arun Pandey, Suresh Pandey, Praveen Srivastava, Kamta Prasad Pandey, Bhudhendra Kumar Jain

**Affiliations:** Sadguru Netra Chikitsalaya, Shri Sadguru Seva Sangh Trust, Jankikund, Satna District, Chitrakoot, Madhya Pradesh, India

**Keywords:** Blindness, effectiveness, refractive services, school vision screening, visual acuity

## Abstract

**Aim:**

To assess the effectiveness of teachers in a vision screening program for children in classes 5th to 12th attending school in two blocks of a district of north central India.

**Materials and Methods:**

Ophthalmic assistants trained school teachers to measure visual acuity and to identify obvious ocular abnormalities in children. Children with visual acuity worse than 20/30 in any eye and/or any obvious ocular abnormality were referred to an ophthalmic assistant. Ophthalmic assistants also repeated eye examinations on a random sample of children identified as normal (approximately 1%, n=543) by the teachers. Ophthalmic assistants prescribed spectacles to children needing refractive correction and referred children needing further examination to a pediatric ophthalmologist at the base hospital.

**Results:**

Five hundred and thirty teachers from 530 schools enrolled 77,778 children in the project and screened 68,833 (88.50%) of enrolled children. Teachers referred 3,822 children (4.91%) with eye defects for further examination by the ophthalmic assistant who confirmed eye defects in 1242 children (1.80% of all screened children). Myopia (n=410, 33.01%), Vitamin A deficiency (n=143, 11.51%) and strabismus (n=134, 10.79%) were the most common eye problems identified by the ophthalmic assistant. Ophthalmic assistants identified 57.97% referrals as false positives and 6.08% children as false negatives from the random sample of normal children. Spectacles were prescribed to 39.47% of children confirmed with eye defects.

**Conclusions:**

Primary vision screening by teachers has effectively reduced the workload of ophthalmic assistants. High false positive and false negative rates need to be studied further.

Population-based studies from India report a prevalence of 2.7% in a rural population and 6.4% in an urban population for uncorrected visual acuity worse than 20/40 in the better eye among children aged 7-15 years.[1,2] The prevalence was 0.78% in a rural population and 0.81% in an urban population for best corrected visual acuity worse than 20/40 in the better eye in the same population.[1,2] Refractive errors were the major cause in 61% of eyes with vision impairment in a rural population and 81.7% of eyes with vision impairment in an urban population.[1,2] Provision of appropriate refractive services and spectacles to children with presenting visual acuity worse than 20/40 was expected to benefit approximately 70% of children within the rural population and over 80% of children in the urban population.[1,2]

Strategies to address eye health of children in India have focused on school eye health programs. School eye screening programs have been part of the activities of the district blindness control society (DBCS) activities since 1996.[3] Additionally, several practitioners in the private and non-government (NGO) sector run school eye screening programs to supplement and complement the activities of the DBCS. Screening of children in schools is most commonly done by trained school teachers although some programs utilize ophthalmic assistants and ophthalmologists for primary screening. Screening school children is arguably the second largest program of the national program for control of blindness in India after cataract surgery and is currently a priority of the Sarva Shiksha Abhiyan education initiative of the government. However, evidence pertaining to school screening programs in India is scarce, especially when compared to evidence for initiatives addressing age-related cataracts.

Sadguru Netra Chikitsalaya (SNC), located at Chitrakoot in Madhya Pradesh provides eye care services to districts bordering Madhya Pradesh, Uttar Pradesh and Bihar in North Central India. SNC has a dedicated pediatric ophthalmology center functional since 2002 to cater to the eye care needs of the pediatric population of these districts. The pediatric ophthalmology center at SNC has an active school eye screening program that covers schools in these districts and uses the services of trained school teachers for this program. In this paper, we report the effectiveness of using teachers for screening for eye disorders among children attending classes 5^th^ to 12^th^ at schools.

## Materials and Methods

The project covered two blocks- Majhghawan and Amarpattan-in Satna district of Madhya Pradesh of central India in the academic year 2007-08. Initially, a list of schools for children studying from the 5^th^ to the 12^th^ standard was prepared in consultation with the district and block level educational authorities. After preparation of the list, details regarding the project were communicated to the principal/head of these schools through a letter and in person. Each principal was requested to nominate at least one teacher for training under the program. Female teachers teaching science subjects and wearing spectacles were preferred for the training but this was not an inclusion criterion for training. An ophthalmic assistant with more than three years experience trained the teachers in groups of 20-25 each so that each teacher could be given individual attention. The training sessions were for a day and included theoretical instruction about common eye diseases and visual acuity, and practical demonstrations on measuring visual acuity. Each trained teacher was provided with a kit that included visual acuity cards (illiterate E 20/30 optotype), a measuring tape for 20 feet distance, a screening card to record details of the child, a referral card for children identified with poor vision or other eye problems and eye health education material.

After completion of the training, the teacher assessed the visual acuity of each child in the school using an illiterate E card (20/30 optotype) at 20 feet distance. The visual acuity of each eye of the child was measured separately with the other eye closed using the palm of the child. Visual acuity measurements were made under normal daylight illumination. The illiterate E card was rotated randomly in different directions and the child had to correctly identify at least four out of five optotypes to be denoted as having good vision. The child was considered to have a visual problem if the child was unable to correctly identify at least four out of five optotypes. Teachers also looked for obvious eye problems like squint, cataract, leucoma, ptosis, Bitot's spots and for symptoms suggestive of underlying ocular conditions like watering of the eyes, redness and headache. The teachers categorized and listed children who were examined as “having good vision and NO OBVIOUS EYE PROBLEM and need no further referral” and “need further referral”.

Trained ophthalmic assistants subsequently screened the children identified as “need further referral” by the school teacher at a date not later than a month from the initial screening by the teacher. The ophthalmic assistants examined children at the school itself or at a central location if children of several schools were examined on the same day. The examination included assessment of presenting and best corrected visual acuity, refraction, and anterior segment examination of the eye as well as testing for ocular motility and alignment. The ophthalmic assistant prescribed spectacles for children identified with refractive errors and referred to SNC children identified with eye problems that needed examination by an ophthalmologist. Children identified as needing spectacles were provided with spectacles free of cost by SNC.

Approximately 1% of children who were identified as normal by the school teacher were also included for examination by the ophthalmic assistants. These children were identified (from a sample frame that consisted of all children identified as normal by the school teachers) using a simple random sampling technique without replacement.

Reports received from each school were entered into a computer in an MS-Office Excel worksheet. The data was cross-verified at the central office level and records with incomplete or inconsistent data were not considered for analysis. We analyzed data focusing on several monitoring indicators including the coverage, quality of training, and the organization and quality of refraction services. Data on false negatives was derived from the examination by ophthalmic assistants of children identified as normal by the teacher. Data on false positives was derived from the examination by ophthalmic assistants of children identified as having an eye problem by the teacher.

## Results

The project was carried out from August 1, 2007 to March 31, 2008. Five hundred and thirty teachers from 530 schools in two blocks (one teacher per school) were trained under the project. The project enrolled 77,778 children studying in the 5^th^ to 12^th^ standard. Teachers screened 68,833 of these 77,778 enrolled children achieving coverage of 88.50%. On an average, a teacher in the project screened 130 children. The evaluation of the project based on some monitoring indicators is presented in Table 1.

**Table 1:** Monitoring and evaluation indicators for school eye screening programs

Parameter	Indicator	Benchmark	Project Performance
Number of schools covered			530
Number of teachers trained	Coverage	At least 1 teacher per school	530
Proportion of enrolled children screened	Coverage	80-100%	88.50%
Number of children with poor vision referred to OA*	Quality of screening by teachers	5-10%	4.91%
Number of children examined by OA*	Organization of refraction services	60-90%	77.32%
Number of spectacles prescribed	Quality of refraction services	40-80%	39.47%

* OA – Ophthalmic assistant

Teachers identified and referred 3,822 children (4.91%) with eye defects for further examination by the ophthalmic assistant. Ophthalmic assistants screened 2,955 (77.32%) of these 3,822 children and confirmed eye defects in 1242 children (1.80% of all screened children). The diagnosis of children identified with eye defects by the ophthalmic assistant is presented in Table 2. One thousand seven hundred and thirteen (57.97%) children were identified as false positives after the examination by the ophthalmic assistants. Ophthalmic assistants examined another 543 children who were identified as normal by the school teachers and identified 33 (6.08%) of these 543 children as false negatives. Two of these 33 false negatives were bilateral blind with visual acuity worse than 20/200 in both eyes. Eighteen of the 33 children identified as false negatives had myopia, four children had strabismus, four children had vitamin A deficiency, three children had ptosis, two children had coloboma of the iris and hypermetropia and amblyopia were detected in one child each.

**Table 2:** Diagnosis of children identified as true positives in the program

Diagnosis	N	% among all children identified with eye defects by OA*	% among all screened school children
Myopia	410	33.01	0.60
Hypermetropia	61	4.91	0.09
Amblyopia	35	2.82	0.05
Cataract	33	2.66	0.05
Cataract surgery	4	0.32	0.01
Strabismus	134	10.79	0.19
Ptosis	24	1.93	0.03
Vitamin A deficiency	143	11.51	0.21
Ocular Infections	72	5.80	0.10
Others	326	26.25	0.47

* OA-Ophthalmic Assistant

## Discussion

The project covered nearly 90% of enrolled children and achieved a target of training at least one teacher per school. The coverage is an indicator of the organization of the entire program and the ability to involve schools and teachers with the program. The quality of screening by teachers can be assessed by the number of referrals made to an ophthalmic assistant. The number of spectacles prescribed to children referred to the ophthalmic assistant by the teachers is another indicator of the quality of screening by teachers since most of the referrals are made based on a visual acuity cutoff and aim at identifying refractive errors. Limburg *et al*.. reported average referral rates to an ophthalmic assistant of 5.0% (range 1.8-29.4%) and a spectacle prescription rate of 40-80%.[3] He suggested that referral rates outside of 5-10% and spectacle prescription rates lower than 40% may indicate the need to evaluate the training of teachers. The number of children referred to an ophthalmic assistant (4.91%) and the number of children prescribed spectacles (39.47%) compare favorably with the monitoring and evaluation benchmarks proposed by Limburg *et al*.[3] The number of children examined by the ophthalmic assistant after referral from the teachers is an indicator of the organization of refractive services and the ability of teachers to follow up on referrals. Referral systems need to be checked if less than 50% of children referred are examined by the ophthalmic assistant. The number of children examined by the ophthalmic assistant (77.32%) in our program compares favorably with the benchmark proposed by Limburg *et al*. (60-90%).[3]

The screening program can also be assessed by the true positives, the false positives and false negatives. The diagnostic distribution of true positives in our project is not dissimilar to other reports from India with uncorrected refractive errors, the major ocular morbidity in this age group.[4–6] Children identified as having abnormalities by the teacher but confirmed as having no abnormalities by the ophthalmic assistant are considered as false positives. The false positive rate is important as it is a measure of over-diagnosis/over-referrals by teachers. Such over-diagnosis and over-referrals have implications for the eye care system (the ophthalmic assistant has to additionally screen these children when they really need not be screened adding a time and cost component to the screening program), for children (children who are normal are labeled as not normal and may induce some anxiety in children and their caretakers) and for caretakers (caretakers of children identified as not normal by the teachers but later confirmed as normal by ophthalmic assistants may lose faith in the process or ability of the teacher to identify abnormalities). Reducing the false positives will reduce the workload on the ophthalmic assistant besides reducing the costs of the screening program. Children identified as normal by the teachers but confirmed with abnormalities by the ophthalmic assistant are considered as false negatives. The false negative rate is important as it indicates how many abnormal children are missed by the teachers and reflects the quality of training to teachers. School eye screening programs will want a low false negative rate as these programs may be the only avenue for eye exams for many of these children.

The high false positive rate (57.97%) in this program indicates that nearly two-thirds of referrals to the ophthalmic assistant were not necessary. The high false positive rates can be due to a mix of the visual acuity cutoff used (20/30) and an inclination of teachers to err on the side of caution and refer in case of any doubt. The false negative rate in the project (6.08%) is reasonably good supporting the premise that teachers were not referring to the ophthalmic assistant only when they were sure that the child was normal. It is a moot point if the current program has to be tweaked to improve the false positive and false negative rate. Reducing the false positive rate is definitely advantageous since it will reduce the workload of the ophthalmic assistant and the costs of the program. Reducing the false negative rate further will ensure that children who need care are not missed out. The program can look at three options to reduce the false positive rates. One option is to improve the quality of training to teachers and make them more confident such that the over-referrals are reduced while children with abnormality are not missed. This may need more intensive training within the one-day period. Increasing the duration of the training may not be feasible since many schools have only one teacher and the school has to remain closed for the duration of training. An alternate option is to train teachers during the vacation when schools are closed; however, this will cut into the vacation time of the teacher and may not be popular. Increasing the size of the E optotype from the present 20/30 to 20/40 or 20/60 may help to reduce the false positives. Increasing the cutoff may also help limit identification of children with significant refractive errors and aid prioritization of service delivery, especially when healthcare resource allocations are coming down each year. The impact of a strategy that reduces the visual acuity cutoff to 20/40 or 20/60 needs to be studied based on yield, costs, and the impact on the children who now will be labeled as normal although they may have mild refractive errors. A third option is to utilize ophthalmic assistants for screening all children, however, this option is not feasible considering the lower than needed number of ophthalmic assistants/optometrists in India and the number of children to be screened.[7,8]

There are concerns whether the program adds substantially to the existing workload of teachers and may hinder their primary responsibility of teaching. On an average, each teacher screened about 130 children in a year and referred about seven children for further examination to the ophthalmic assistant. We estimate that each teacher will have to spend about 10-12 h (assuming 5 min on an average for examining each child) to screen children plus an additional 8 h for training, a total of maybe 20 h in a year for this program. There were however substantial benefits to primary screening by school teachers. The workload of ophthalmic assistants reduced significantly; the ophthalmic assistants needed to screen only about 5% of all school children. Additionally, only a very small proportion of abnormal children (6.08%) were missed through the program. Considering the benefits from utilizing teachers, eye care programs may want to design strategies to sustain the interest of the teachers in the program.

Overall, our results indicate that utilizing the services of teachers for screening the eyes of school-going children from classes 5^th^ to 12^th^ substantially reduces the workload of eye care service providers. A major lacunae in the program is the ability to reach out to children not attending schools, however, this may be addressed by strategies that aim to improve universal education. Studies that provide evidence on strategies to reduce false positive and false negative rates and the frequency of school screening (annual or at specific time periods) will help improve the effectiveness of the screening program.
